# Prevalence of the Equol-Producer Phenotype and Its Relationship with Dietary Isoflavone and Serum Lipids in Healthy Chinese Adults

**DOI:** 10.2188/jea.JE20090185

**Published:** 2010-09-05

**Authors:** Baohua Liu, Liqiang Qin, Aiping Liu, Shigeto Uchiyama, Tomomi Ueno, Xuetuo Li, Peiyu Wang

**Affiliations:** 1 Department of Social Medicine & Health Education, School of Public Health, Peking University, Beijing, PR China; 2 Department of Nutrition and Food Hygiene, School of Radiation Medicine and Public Health, Soochow University, Suzhou, PR China; 3 Saga Nutraceutical Research Institute, Otsuka Pharmaceutical Co., Ltd., Yoshinogari, Kanzaki, Saga, Japan; 4 Otsuka (China) Investment Co., Ltd., Beijing, PR China

**Keywords:** equol, isoflavone, soy, serum lipids

## Abstract

**Background:**

Studies have suggested that daidzein-metabolizing phenotypes have beneficial effects on a range of health outcomes. We investigated the prevalence of equol producers and the relationship of equol phenotype with habitual isoflavone consumption and serum lipid concentrations in 200 Chinese adults in Beijing.

**Methods:**

After the baseline survey and dietary records, 200 healthy adults in Beijing were challenged with a soy-isoflavone supplement for 3 days; 24-hour urine samples were collected before and after the challenge. Isoflavones and their metabolites in urine were measured to determine equol phenotype. Serum lipids, uric acid, and other biochemical markers were also measured.

**Results:**

Only 26.8% of the participants excreted equol when on a regular diet, as compared with 60.4% after the challenge. After the challenge, urinary isoflavonoid excretion increased in all participants, while equol excretion increased only in equol producers. Isoflavone intake was correlated with urinary isoflavone (range *r* = 0.49–0.58, *P* < 0.01). As compared with nonproducers, equol producers were less likely to consume cereals (*P* < 0.001). There was no significant correlation between serum lipids and isoflavone intake. Serum lipids were not significantly affected by equol phenotype.

**Conclusions:**

Urinary equol excretion was detected in about 25% of participants under their usual dietary conditions. Their potential to produce equol was increased after the challenge. Urinary isoflavone levels may serve as a useful biomarker for isoflavone intake in populations. We observed an association between equol phenotype and cereal intake. Our findings also suggest that dietary isoflavone intake has no significant effect on serum lipids in healthy participants, regardless of equol phenotype.

## INTRODUCTION

Isoflavones contained in soybeans (soy isoflavones) possess estrogen-like activity^1^ and protect against climacteric disorders, osteoporosis, and hyperlipidemia.^2^^–^^4^ These effects are apparent in some populations, but not in others.^5^^,^^6^ This variation may be due to differences in the ability to produce equol, which is a key metabolite of daidzein and an isoflavone found in most soy foods.^7^^–^^9^ Equol is believed to be the active form of soy isoflavones and has a stronger estrogen-like activity than daidzein. Thus, high equol producers may have greater protection against hormone-dependent diseases than do low equol producers.^10^

The prevalence of equol producers has been reported to be 20% to 35% among Western adults who consume soy foods or isoflavone supplements.^4^^,^^11^^–^^14^ In contrast, the prevalence has been reported to be as high as 50% to 55% in adults living in Asia.^15^^–^^19^ The reason for this disparity is most likely differences in the macrocomposition of the diets consumed in these areas.^4^

To the best of our knowledge, no study has reported the prevalence of equol producers in China. We therefore conducted an epidemiological study in Beijing, China, in November 2007 to determine the physiological range of 24-hour urinary equol excretion in people eating their usual diet, as well as the prevalence of equol producers after a soy-isoflavone challenge. We also evaluated the association of habitual isoflavone consumption and equol production status with levels of serum lipids and uric acid.

## METHODS

### Study participants

Healthy adults who had lived in Beijing for more than 5 years were recruited from communities throughout Beijing. Interviews were conducted by telephone and in person to identify qualified participants. Participants were excluded if they had a disease of the digestive system, were pregnant, or had received hormone therapy or antibiotics within the past month. Candidates underwent a health screen to confirm health status. One hundred men and 100 women in 4 age groups (20–29, 30–39, 40–49, and 50–75 years) of 25 participants each were enrolled in the study. The occupations of the participants included university student, university teacher, manual worker, company employee, driver, doctor and nurse of community hospitals, and retired person. This study was approved by the medical ethics committee of Peking University. The participants were informed of the requirements of the study and the experimental protocols, and all provided written informed consent.

### Study protocol

#### Baseline survey

All participants were interviewed by trained interviewers and general information (age, sex, smoking status, alcohol consumption, medication use, diet, medical history, and general physical condition; Figure) was collected by using a questionnaire at the first visit. In addition, the participant’s physical condition, daily food intake, and medication use were recorded for the previous day and the day of the 24-hour urine collection. A booklet with details on serving sizes was given to participants to increase the accuracy of food intake record. This included pictures of some commonly consumed foods in different portion sizes, a ruler, a thickness guide, and a serving dish size guide. Completed questionnaires and dietary records were sent to our laboratory. Participants were also given labeled collection bottles for storing 24-hour urine samples. Samples were immediately sent to our laboratory for measurement of volume, then stored in sealed 10-mL plastic containers at −80°C until analysis. After the urine collection, 10-mL samples of fasting venous blood were collected in 2 EDTA-containing tubes. The sera were separated by centrifugation at 3000 rpm for 10 minutes at 4°C and stored at −20°C. In premenopausal women, fasting blood samples were collected on the 11th day of the menstrual cycle. Serum glucose, total protein, albumin, total cholesterol (TC), triglyceride (TG), high-density-lipoprotein cholesterol (HDL-C), low-density-lipoprotein cholesterol (LDL-C), creatinine, blood urea nitrogen (BUN), uric acid, γ-glutamyltranspeptidase (γ-GT), glutamate-oxaloacetate transaminase (GOT), and glutamic-pyruvic transaminase (GPT) were measured using an automated biochemical analyzer (Type7170A HITACHI, Japan) at Peking University Third Hospital, Beijing, China.

**Figure.**
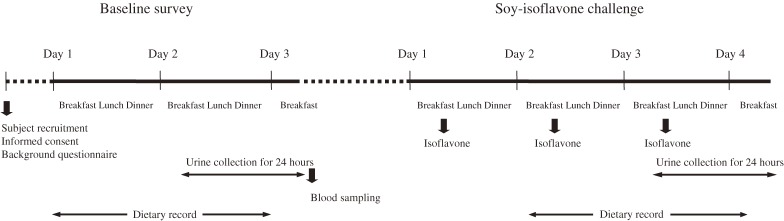
Study protocol

#### Soy-isoflavone challenge

After the baseline survey, the participants were challenged with a soy-isoflavone supplement (capsule) at a dose of 41 mg soy-isoflavone aglycone per day for 3 consecutive days. On day 2 of the challenge, the participant’s physical condition, daily food intake, and medication use were recorded as described in the baseline survey; 24-hour urine samples were collected on the third day of the challenge. Supplement compliance was also investigated. Participants were asked to maintain their usual lifestyle, but to avoid the consumption of alcohol during the entire study period.

#### Analysis of urine samples

Soy-isoflavone and its metabolites in urine were assayed by Saga Nutraceuticals Research Institute, Otsuka Pharmaceutical Co Ltd, Japan, using HPLC with a type L-5030 column and an SPD-10AVP UV-VIS detection system (Capcell PACK UG 120 5 µm 4.6Φ × 250 mm; Shiseido Co Ltd, Japan).^20^ Daidzein, genistein, equol, and β-glucuronidase were purchased from Sigma Chemical Company (St. Louis, MO, USA). All other chemicals and solvents used for HPLC were analytical-grade reagents purchased from Wako Pure Chemical Industries (Osaka, Japan). Soyaflavone (the standard) was generously donated by Fuji Oil (Osaka, Japan). Data processing was carried out with CoulArrayTM software (ESA Inc). The amounts of soy-isoflavone and its metabolites were expressed on the basis of 24-hour total urine volume. The detection limit for equol was 0.68 nmol/mL. Participants with detectable equol in their urine were classified as equol producers; those below the detection limit were classified as non-equol producers.

#### Estimation of soy protein and isoflavone intake from 2-day dietary records

The intake of dietary nutrients and isoflavones during the survey period was assessed using the Chinese Food Composition Table^21^ and Microsoft Excel 2003. Total isoflavone intake was calculated using the following formula: total isoflavone intake per day = [Σ(amount of soy food noted in two 2-day dietary records × isoflavone level in this amount of soy food)]/4. Because soy foods contain different volumes of water, total soy protein is a better index to indicate the total consumption of soy food. We used the formula: total soy protein per day = [Σ(amount of soy food noted in two 2-day dietary records × protein quantity in this amount of soy food)]/4.

### Data analysis

All values are presented as means ± standard deviations (SD). Urinary excretion of isoflavonoids was compared at baseline and after challenge with the Wilcoxon signed-rank test and between equol producers and nonproducers with the Mann-Whitney U test. The data for HDLC, TG, γ-GT, GOT, and GPT were log-transformed for analyses because they were skewed. The validity of the 2-day dietary records was evaluated by calculating the Spearman correlation coefficients of isoflavone intakes derived from 2-day dietary records and urinary isoflavone excretion. Differences in demographic/lifestyle factors and nutrient intake between equol producers and nonproducers were analyzed using the *t* test or, when data were not normally distributed, the Mann-Whitney U test for continuous data and the chi-square test or Fisher’s exact test for categorical data. Binary logistic regression analyses were performed to assess relationships between equol phenotype and dietary factors. Selection of variables to be considered for inclusion in the regression models was based on a priori knowledge of factors that have previously been shown to be associated with dietary factors and equol-producer phenotype. Only those variables that were significantly associated with equol phenotype in univariate analyses were included in the final models. Because of its skewed distribution, isoflavone intake was divided into 2 groups (high and low isoflavone intake) by using median intake. We then used binary logistic regression to evaluate the relationship between isoflavone intake and serum biochemical markers, serum lipids, uric acid, and other biochemical markers, after adjusting for potential confounding factors. Potential confounding factors included in the regression model were those variables that were significantly associated with isoflavone intake in univariate analyses. The associations of serum lipids, uric acid, and other biochemical markers with isoflavones were also estimated in the equol phenotype subgroup. All analyses were performed using SPSS version 10.0 (SPSS Inc., Chicago, IL), and a *P* value ≤0.05 was considered statistically significant.

## RESULTS

### Prevalence and urinary isoflavonoid excretion of equol producers on a regular diet

Of the 100 men and 100 women recruited, all completed the health and demographics questionnaire and two 2-day dietary records. During the baseline survey, 17 participants (7 men and 10 women) were excluded because they did not comply with the study protocol for personal reasons. Thus, 183 participants were included in the analysis of urinary equol excretion while on their regular diets. The mean age of these participants was 40.4 ± 12.5 years. The mean (± standard deviation [SD]) height, weight, and BMI were 1.66 ± 0.08 m, 64.92 ± 11.41 kg, and 23.52 ± 3.10 kg/m^2^, respectively.

Equol excretion was noted in 49 of the 183 participants (26.8%, 95% CI: 20.4%–33.2%) who were on regular diets and returned the 24-hour sample. Among the 93 male participants, 25 (26.9%) excreted equol; 24 (26.7%) of the 90 female participants produced equol. The proportion of equol producers did not significantly differ between men and women (*P* > 0.05).

Among those on a regular diet, daily urinary excretion of equol was higher and excretion of daidzein was lower in equol producers than in nonproducers (equol phenotype was determined by soy-isoflavone challenge, as below, *P* < 0.05; Table 1). There were no significant differences between producers and nonproducers in the excretion of other isoflavonoids.

**Table 1.** 24-hour urinary isoflavonoid excretion by equol phenotype in participants on a regular diet (µmol/24 hours)

Urinary isoflavone	Equol producers (*n* = 104)^a^		Nonproducers (*n* = 76)^a^		*P*^b^
	
	Mean ± SD	Range	Mean ± SD	Range	
Equol	5.75 ± 11.02	0–76.56	0.35 ± 1.99	0–15.56	<0.001
Daidzein	8.24 ± 10.70	0–52.72	12.53 ± 12.00	0–54.77	0.004
Dihydrodaidzein	3.04 ± 5.09	0–34.47	4.21 ± 6.52	0–32.73	0.43
O-DMA^c^	1.81 ± 2.76	0–76.56	3.42 ± 5.61	0–32.32	0.09
Genistein	4.09 ± 5.48	0–26.50	5.15 ± 5.69	0–28.54	0.08
Dihydrogenistein	3.74 ± 8.34	0–63.44	2.64 ± 4.45	0–42.54	0.35
Glycitein	1.63 ± 2.14	0–9.57	2.19 ± 2.56	0–9.76	0.16
Dihydroglycitein	0.01 ± 0.09	0–0.92	0.02 ± 0.20	0–1.76	0.82
Total isoflavone^d^	26.27 ± 31.92	0–148.31	30.17 ± 30.07	0–115.13	0.12

^a^Determined by soy-isoflavone challenge.

^b^Equol producers and nonproducers compared using Mann-Whitney U test.

^c^O-DMA: O-Desmethylangolensin.

^d^Total isoflavone = daidzein plus its metabolites dihydrodaidzein, equol, O-desmethylangolensin; genistein plus its metabolite dihydrogenistein; and glycitein plus its metabolite dihydroglycitein.

### Prevalence and urinary isoflavonoid excretion of equol producers after the soy-isoflavone challenge

After the soy-isoflavone challenge, 3 males did not comply with the protocol and were excluded from the analyses. Thus, 197 participants were included in the equol-production analyses in the study. The mean age (± SD) of these participants was 40.0 ± 12.5 years, and mean height, weight, and BMI were 1.66 ± 0.08 m, 64.80 ± 11.38 kg, and 23.52 ± 3.10 kg/m^2^, respectively.

The percentage of equol producers increased to 60.4% (95% CI: 53.6%–67.2%) among the 197 participants. Among the 97 male participants, 56.7% (46.8%–66.6%) were equol producers. Among the 100 female participants, 64.0% (54.6%–73.4%) were equol producers. The proportion of those secreting equol after the challenge was also not significantly different between men and women (*P* > 0.05).

There were no differences between the proportions of equol producers and nonproducers among current or former smokers (16% vs 15%, *P* = 0.91) or between those who did and did not consume alcohol (14% vs 19%, *P* = 0.36). However, the difference in BMI between equol producers and nonproducers was of borderline statistical significance (23.2 ± 3.2 kg/m^2^ vs 24.1 ± 3.0 kg/m^2^, *P* = 0.05). No other significant associations were found (Table 2).

**Table 2.** Demographic and lifestyle characteristics of study participants by equol phenotype

	Equol producers^a^ (*n* = 119)	Equol nonproducers^a^ (*n* = 78)	*P*^b^
Age (years)	38.8 ± 12.1^c^	41.8 ± 12.9	0.11
Sex [*n* (%)]			
Male	55 (46)	42 (54)	0.30
Female	64 (54)	36 (46)	
Height (m)	1.66 ± 0.08	1.66 ± 0.07	0.94
Weight (kg)	63.8 ± 11.2	66.4 ± 11.6	0.12
Body mass index (kg/m^2^)	23.2 ± 3.0	24.1 ± 3.2	0.05
Education, years [*n* (%)]			
≤12	32 (27)	25 (32)	0.24
13–15	15 (13)	16 (21)	
16	34 (29)	15 (19)	
≥17	38 (32)	22 (28)	
Smoker [*n* (%)]			
Current or former	19 (16)	12 (15)	0.91
Never	100 (84)	66 (85)	
Alcohol drinker [*n* (%)]			
Yes	17 (14)	15 (19)	0.36
No	102 (86)	63 (81)	

^a^Determined by soy-isoflavone challenge.

^b^Determined by *t* test or chi-square test.

^c^Mean ± SD (all such values).

The soy-isoflavone challenge significantly increased the urinary excretion of isoflavonoids in both groups, while the urinary excretion of equol increased only in equol producers (*P* < 0.05). The higher rate of equol excretion resulted in a higher rate of excretion of total isoflavone in the equol producers, although excretions of daidzein, dihydrodaidzein, and O-desmethylangolensin (O-DMA) were lower in equol producers than in nonproducers (Table 3).

**Table 3.** 24-hour urinary isoflavonoid excretion by equol phenotype after a soy-isoflavone challenge (µmol/24 hours)

Urinary isoflavone	Equol producers (*n* = 104)^a^		Nonproducers (*n* = 76)^a^		*P*^b^
	
	Mean ± SD	Range	Mean ± SD	Range	
Equol	35.16 ± 21.76	1.84–121.12	not detected	not detected	<0.001
Daidzein	50.22 ± 22.15	2.89–103.19	58.01 ± 21.87	1.47–116.63	0.01
Dihydrodaidzein	13.97 ± 10.87	0–61.05	23.27 ± 16.91	0–71.68	<0.001
O-DMA^c^	7.18 ± 6.29	0–36.04	11.10 ± 11.25	0–54.94	0.03
Genistein	8.12 ± 6.64	0–37.98	8.26 ± 7.71	0.37–60.35	0.74
Dihydrogenistein	3.49 ± 7.88	0–64.62	2.59 ± 4.40	0–21.32	0.39
Glycitein	23.61 ± 9.92	0–47.96	22.54 ± 9.32	0.51–50.84	0.24
Dihydroglycitein	0.46 ± 0.99	0–4.32	0.56 ± 1.38	0–7.26	0.96
Total isoflavone^d^	142.20 ± 54.09	14.19–319.56	126.34 ± 48.67	2.35–306.75	0.04

^a^Determined by soy-isoflavone challenge.

^b^Equol producers and nonproducers compared using Mann-Whitney U test.

^c^O-DMA: O-desmethylangolensin.

^d^Total isoflavone = daidzein plus its metabolites dihydrodaidzein, equol, O-desmethylangolensin; genistein plus its metabolite dihydrogenistein; and glycitein plus its metabolite dihydroglycitein.

### The effect of dietary habits on equol producers

The validity of the 2-day dietary records was assessed by comparing them with urinary isoflavone measurements of participants on a regular diet. As shown in Table 4, the Spearman correlation coefficients for total isoflavone intake and its major components ranged between 0.50 and 0.60 (*P* < 0.01), suggesting that the validity of the 2-day dietary record was acceptable for assessing dietary isoflavone intake.

**Table 4.** Spearman correlation coefficients for isoflavone intake on 2-day dietary records and mean of urinary isoflavonoid measurements among participants on a regular diet

Dietary intake (mg/day)		Urinary excretion (µmol/24 hours)		*r*
Daidzein	7.57 ± 12.12	Total daidzein^a^	19.79 ± 22.93	0.55^e^
Genistein	10.18 ± 17.38	Total genistein^b^	6.42 ± 9.69	0.60^e^
Glycitein	1.05 ± 2.72	Total glycitein^c^	1.98 ± 2.85	0.50^e^
Isoflavone	18.80 ± 31.96	Total isoflavone^d^	28.18 ± 31.85	0.58^e^

^a^Total daidzein = daidzein plus its metabolites dihydrodaidzein, equol, and O-desmethylangolensin.

^b^Total genistein = genistein plus its metabolite dihydrogenistein.

^c^Total glycitein = glycitein plus its metabolite dihydroglycitein.

^d^Total isoflavone = total daidzein + total genistein + total glycitein.

^e^*P* < 0.01.

The most commonly consumed soy foods among the participants were soy milk and tofu. No differences were found in the consumption of any type of soy food, total soy foods, or soy protein between the 2 equol phenotype groups. Daily dietary isoflavone intakes were also similar between equol producers (19.1 ± 22.3 mg/day) and nonproducers (17.3 ± 17.1 mg/day) (*P* > 0.05) (Table 5).

**Table 5.** Daily intakes of nutrients, food groups, and isoflavone (means ± SD) by equol phenotype according to 2-day dietary records

Dietary measure	Equol producers^a^ (*n* = 119)	Equol nonproducers^a^ (*n* = 78)	*P*	

			Unadjusted^b^	Adjusted^c^
Nutrients				
Energy (kcal)	2081 ± 524	2161 ± 468	0.16	0.62
Fat (g)	64 ± 25	62 ± 23	0.47	0.65
Carbohydrate (g)	309 ± 81	336 ± 78	0.03	0.96
Protein (g)	80 ± 26	78 ± 20	0.80	0.19
Animal protein (g)	32 ± 18	29 ± 14	0.10	0.26
Vegetable protein (g)	48 ± 16	50 ± 13	0.20	0.34
Total soy protein (g)	6 ± 7	5 ± 5	0.90	0.58
Total dietary fiber (g)	13 ± 8	13 ± 7	0.99	0.74
Calories from fat (%)	27 ± 7	25 ± 6	0.02	0.59
Calories from carbohydrate (%)	58 ± 7	61 ± 7	0.01	0.29
Calories from protein (%)	15 ± 2	14 ± 7	0.01	0.14
Food groups (g/d)				
Soybeans	1.3 ± 3.9	1.3 ± 3.6	0.88	0.64
Soy milk	43.2 ± 84.2	31.9 ± 63.2	0.61	0.23
Tofu	22.0 ± 31.6	23.5 ± 29.9	0.60	0.79
Other soy foods	12.7 ± 23.8	10.9 ± 18.1	0.94	0.40
Total soy foods	79.2 ± 92.5	67.7 ± 71.1	0.75	0.24
Vegetables	219 ± 134	244 ± 131	0.10	0.56
Tubers	48 ± 57	49 ± 54	0.59	0.70
Fruit	187 ± 157	171 ± 151	0.44	0.64
Cereals and cereal products	262 ± 105	313 ± 101	<0.001	0.001
Meat and meat products	75 ± 56	63 ± 46	0.22	0.11
Poultry and poultry products	32 ± 49	19 ± 30	0.03	0.12
Milk and milk products	119 ± 127	112 ± 135	0.56	0.88
Eggs and egg products	42 ± 39	41 ± 35	0.97	0.43
Fish, shellfish, and mollusks	26 ± 33	32 ± 42	0.49	0.09
Isoflavone intake (mg/day)				
Daidzein	7.8 ± 8.7	7.0 ± 6.8	0.65	0.57
Genistein	10.4 ± 12.1	9.4 ± 9.3	0.65	0.63
Glycitein	1.0 ± 1.7	0.9 ± 1.2	0.96	0.86
Total isoflavone	19.1 ± 22.3	17.3 ± 17.1	0.68	0.62

^a^Determined by soy-isoflavone challenge.

^b^Determined by Mann-Whitney U test.

^c^Adjusted variables that were included in the cereal and cereal-product model were BMI, energy intake, poultry, and poultry products. Adjusted variables that were included in the poultry and poultry-product model were BMI, energy intake, cereals, and cereal products. Adjusted variables included in other models were BMI, energy intake, cereals and cereal products, and poultry and poultry products.

In unadjusted analyses, equol producers were found to consume more poultry and less carbohydrate, and consequently obtained fewer calories from carbohydrate (*P* < 0.05). However, these differences disappeared after adjustment for BMI, energy intake, consumption of cereals and cereal-based products, and consumption of poultry and poultry-based products (*P* > 0.05). Analyses of the data on food groups showed that cereals and cereal-based products were consumed less by equol producers than by nonproducers (262 ± 105 vs 313 ± 101 grams/day, *P* = 0.001). This difference changed little after adjustment (Table 5).

### Effects of soy-isoflavone consumption on serum lipids and other biochemical markers, by equol phenotype

The median intake of isoflavones among the participants—14.3 mg/day—was defined as the cut-off value between low and high isoflavone intakes. The median isoflavone intakes for participants on a regular diet were 4.6 mg/day and 23.6 mg/day in the low-isoflavone-intake and high-isoflavone-intake groups, respectively.

The participants with high isoflavone intake had somewhat higher BMIs, and consumed more energy, protein, fat, fiber, vitamin A, thiamine, vitamin E, calcium, phosphorus, magnesium, iron, zinc, copper, and manganese (data not shown).

As shown in Table 6, after adjustment for confounding factors, no significant differences were found for TC, TG, HDLC, LDLC, or other biochemical markers between the 2 groups of participants classified by habitual isoflavone intake (*P* > 0.05). The results did not change markedly in the analyses of equol phenotype subgroups (*P* > 0.05).

**Table 6.** Comparison of serum biochemical markers in low and high isoflavone consumers, by equol phenotype^a^

	All subjects^b^		Equol nonproducers^c,e^		Equol producers^d,e^	
			
	Low isoflavone intake (*n* = 98)	High isoflavone intake (*n* = 99)	Low isoflavone intake (*n* = 36)	High isoflavone intake (*n* = 42)	Low isoflavone intake (*n* = 62)	High isoflavone intake (*n* = 57)
TC (mmol/L)	4.90 ± 0.91	4.99 ± 0.94	5.02 ± 1.01	4.92 ± 0.83	4.84 ± 0.84	5.05 ± 1.01
TG (mmol/L)	1.44 ± 1.13	1.51 ± 1.14	1.58 ± 1.43	1.78 ± 1.41	1.36 ± 0.91	1.31 ± 0.85
HDL-C (mmol/L)	1.56 ± 0.40	1.57 ± 0.40	1.57 ± 0.43	1.53 ± 0.43	1.56 ± 0.39	1.60 ± 0.38
LDL-C (mmol/L)	2.71 ± 0.78	2.78 ± 0.84	2.77 ± 0.87	2.72 ± 0.72	2.68 ± 0.73	2.82 ± 0.93
Glucose (mmol/L)	4.91 ± 0.48	4.95 ± 0.73	4.90 ± 0.56	5.08 ± 0.79	4.91 ± 0.43	4.86 ± 0.67
GPT (U/L)	19.60 ± 13.23	22.78 ± 15.73	21.42 ± 15.29	22.88 ± 11.73	18.55 ± 11.89	22.70 ± 18.23
GOT (U/L)	18.27 ± 4.62	18.97 ± 7.03	18.69 ± 4.66	19.00 ± 5.35	18.02 ± 4.62	18.95 ± 8.10
γ-GT (U/L)	20.47 ± 14.13	23.29 ± 23.20	21.53 ± 14.56	27.74 ± 29.95	19.85 ± 13.95	20.02 ± 16.11
Total protein (g/L)	75.44 ± 4.17	75.30 ± 3.24	74.97 ± 4.75	75.02 ± 3.26	75.71 ± 3.80	75.51 ± 3.25
Albumin (g/L)	46.61 ± 2.46	46.78 ± 1.83	46.56 ± 2.79	46.60 ± 1.86	46.65 ± 2.27	46.91 ± 1.82
BUN (mmol/L)	5.17 ± 1.08	5.26 ± 1.18	5.42 ± 1.14	5.34 ± 1.25	5.03 ± 1.03	5.20 ± 1.13
Creatinine (µmol/L)	94.42 ± 15.49	95.15 ± 14.44	95.42 ± 15.64	95.43 ± 12.96	93.84 ± 15.50	94.95 ± 15.54
Uric acid (µmol/L)	274.67 ± 99.00	269.01 ± 84.88	301.94 ± 109.80	283.83 ± 85.71	258.84 ± 89.30	258.09 ± 83.33

^a^All values are mean ± SD. Binary logistic regression was used to analyze the data, after adjusting for BMI, and energy, protein, fat, fiber, vitamin A, thiamine, vitamin E, calcium, phosphorus, magnesium, iron, zinc, copper, and manganese intakes. Means are not significantly different between the low and high isoflavone consumers.

^b^The numbers of low and high isoflavone consumers for TC, TG, HDL-C, and LDL-C analyses were 97 and 98, respectively, but 2 men were excluded due to the use of lipid-lowering drugs. The numbers of low and high isoflavone consumers for glucose analyses were 94 and 96, respectively; 4 and 3 subjects, respectively, were excluded due to the use of glucose-lowering drugs.

^c^The numbers of low and high isoflavone consumers for TC, TG, HDL-C, and LDL-C analyses were 36 and 41, respectively, but 1 man was excluded due to the use of lipid-lowering drugs. The numbers of low and high isoflavone consumers for glucose analyses were 34 and 40, respectively; 2 and 2 subjects, respectively, were excluded due to the use of glucose-lowering drugs.

^d^The numbers of low and high isoflavone consumers for TC, TG, HDL-C, and LDL-C analyses were 62 and 57, respectively, but 1 man was excluded due to the use of lipid-lowering drugs. The numbers of low and high isoflavone consumers for glucose analyses were 60 and 56, respectively; 2 and 1 subjects, respectively, were excluded due to the use of glucose-lowering drugs.

^e^Determined by soy-isoflavone challenge.

Abbreviations: TC, total cholesterol; TG, triglyceride; HDL-C, high-density-lipoprotein cholesterol; LDL-C, low-density-lipoprotein cholesterol; GPT, glutamic-pyruvic transaminase; GOT, glutamate-oxaloacetate transaminase; γ-GT, γ-glutamyltranspeptidase; BUN, blood urine nitrogen.

## DISCUSSION

Soy foods are rich in isoflavones, including daidzein and genistein, and are traditionally consumed by Asian populations. Daidzein can be metabolized to equol by intestinal bacteria, and the absorbed metabolites enter the circulation and are excreted in urine.^3^ The condition of isoflavone-metabolizing bacteria in the gut is the main factor responsible for variation in isoflavonoid profiles among individuals.^14^ We observed that equol producers had less urinary excretion of daidzein, dihydrodaidzein, and O-DMA. A possible reason for this is that daidzein can be metabolized to equol by intestinal microflora, which would reduce plasma daidzein concentration and result in a decrease in urinary daidzein excretion in equol producers.^16^

Urinary isoflavones are an indicator of the intake, absorption, and metabolism of isoflavones. Isoflavones are water-soluble and excreted within 12 to 24 hours.^22^^,^^23^ In the US population, the correlations between dietary isoflavones and urinary isoflavones in 24-hour urinary samples were 0.20 for daidzein, 0.25 for genistein, and 0.25 for total genistein plus daidzein,^24^ which are much lower than those observed in our study and in other studies of Chinese and Japanese populations.^25^^,^^26^ This suggests that urinary isoflavones are likely to be a better biomarker for assessing soy isoflavone intake in populations where such consumption is common.

In this study, only 26.8% of participants excreted equol when on a regular diet, but 60.4% of participants were equol producers after the challenge. These proportions were consistent with those reported in other Asian populations,^13^^–^^17^ but higher than those in Western populations.^4^^,^^11^^–^^14^ Although diet has been reported to influence intestinal microbiota,^27^ we observed few associations between equol phenotype and diet as assessed by 2-day dietary records. We did not observe any significant differences in soy intake between equol producers and nonproducers. We also did not detect an association between equol phenotype and nutrients consumed. In contrast, other studies have reported associations between equol production and consumption of low-fat, high-carbohydrate, diets^13^^,^^14^; plant protein^13^; and meat.^24^^,^^26^^,^^28^ However, these associations have not been consistently observed.^27^^–^^30^ The ability to produce equol does not appear to be easily altered by dietary patterns in adults.^31^^,^^32^ Recent studies suggest a higher prevalence of equol production among vegetarians,^33^ which is inconsistent with our findings. Recently, Gardana reported that equol producers consume less fiber, vegetables, and cereals, and more lipids from animal sources,^34^ a finding supported by our results. However, dietary data were not collected at the time of phenotyping for equol-production status. Thus, the differences may have been artifacts, and these associations should be further investigated in future studies.

It has been suggested that equol-production status influences the response to dietary isoflavone supplementation.^4^ In a relatively small retrospective study, Meyer demonstrated that the lipid-lowering effects of soy protein and isoflavones were limited to equol producers.^35^ In our study, there were no significant differences in lipids or other biochemical markers with respect to level of isoflavone intake or equol phenotype subgroup. These findings are consistent with recent intervention trials that found that equol-production status had no role in the effect of soy isoflavone on plasma lipids.^36^^,^^37^

This study had a number of strengths. First, we evaluated the prevalence and potential determinants of the equol phenotype in participants, using a standardized phenotype protocol, and the 3-day soy-isoflavone challenge ensured sufficient exposure to daidzein, so as to reveal the phenotype accurately. Second, identical inclusion and exclusion criteria were used in the recruitment of all participants. Third, equol phenotype was classified on the basis of total urinary equol output over 24 hours, rather than by urinary equol concentration. Thus, any misclassification would have been similar among the participants. There were, however, some limitations. Assessment of dietary intake is notoriously difficult.^38^ Although detailed dietary information was collected using two 2-day dietary records, we may not have captured the time period of exposures that were associated with the equol phenotype. However, this is unlikely to have affected our findings, given that the phenotype appears to be stable in individuals over time.^18^^,^^39^ In general, it does not seem possible to change phenotype by dietary intervention.^31^^,^^32^

In summary, the equol-production phenotype was exhibited by 60.4% of the participants after a soy-isoflavone challenge. We found no indication that habitual consumption of soy foods was associated with equol phenotype. However, we did observe an association between equol phenotype and cereal intake. Urine isoflavone levels may serve as a valuable biomarker for assessing soy-isoflavone intake in populations where consumption of soy foods is common. Our findings also suggest that dietary isoflavone intake has no significant effect on serum lipids in healthy participants, regardless of equol phenotype.
